# Clinique Chirurgical de l'Hôpital de la Pitié

**Published:** 1843-10-01

**Authors:** 


### Clinique Chirurgicale de l'Hopital de la Pitié. Par *J. Lisfranc*. T. 3. 1843.

This, the concluding volume of M. Lisfranc's Clinical Surgery, falls far short of its predecessors in the amount of valuable practical information and original remark which it contains, while it surpasses them in the endless repetitions, the pompous diction, and the self-sufficiency by which it is disfigured. Treating almost exclusively, of the affections of the female organs of generation, it enters into details too familiar to the English reader to justify our giving a full analysis of its contents; we will merely extract some portions from the various chapters which seem most deserving of attention.

*Polypi of the Uterus.*—M. Lisfranc prefers removing these by excision to the ligature. The operation is attended with little pain, and is seldom followed by any accident. Hæmorrhage is very rare, for in 165 cases he has only met with it twice, while often but a few drops of blood flow. Many inconveniences attend the ligature. Its application is often very difficult and imperfect. Portions of the substance of the uterus are sometimes embraced within its noose, especially when it is applied to polypi having little or no pedicle. It may be displaced, and is often long in becoming detached. Abundant and fetid discharges are caused by its use. So trifling are the sufferings frequently after excision that the practitioner himself is thrown sometimes off his guard, and thus serious or fatal metro-peritonitis may be the consequence of insufficient caution in diet, bodily exercise, &c. Time and hygienic precautions are also required for the subsidence of the hypertrophy of the uterine structure which so commonly accompanies polypus.

Most women, prior to the removal of the polypi, have suffered for a greater or less period from discharges of blood, and will be found liable to congestions of important viscera, unless precautionary bleedings be practised for some time subsequent to the operation. Some patients, indeed, suffer a visible deterioration of health, without any visceral congestion, or anormal condition of the uterine functions, to account for it. An issue on the arm is here indispensable, and of admirable efficacy. It should not be placed on the leg, as risking the production of pelvic excitement.

*Polypi of the Vulva.*—A prolongation or tumefaction of the mucous membrane of the *urethra* is sometimes mistaken for this, forming, as it then does, a large tumor of a reddish or deep red colour. By pressure, however, it is usually found returnable into the canal, and on the passage of a catheter it then offers scarcely any obstruction. Its diagnosis is more difficult when it has become hardened and thickened, and is of less consequence, for if irreducible its removal will become as necessary as that of a polypus. If not indurated it will sometimes yield to antiphlogistics and the use of the douche. When returned, the internal surface of the urethra should be scarified, an operation which is beneficial not only by the disgorgement of the vessels it produces, but by the contractions ensuing upon the cicatrices that are formed. In the absence of inflammation, the best means of all is to touch the part lightly, every few days, with the nitrate of silver.

Polypi of the vulva are usually reddish in colour and soft in consistence. In their removal the knife is to be preferred to the ligature, as their roots usually extend far farther than their apparent insertion, and when they are merely cut off at this point they are very liable to return, a circumstance the author has never known to occur when a careful dissection of their roots has been accomplished.

*Polypi of the Vagina.*—These are far less common than polypi of the uterus. It is very important to bear in mind, that when the polypus has acquired a certain volume, it exerts traction upon the part whereto it is attached, and, in this way, often draws down the rectum or vesico-rectal septum to such an extent, that, without great care, these parts may become perforated during the operation. And this accident is the more likely to happen, because, in pursuing the dissection of the roots of the polypus, these will often be found extending into the very substance of the vagina and rectum. Where the complete removal of the disease is impracticable, a slight application of the proto-nitrate of mercury should be made to the portion which remains.

*Moles.*—"A mole is a fleshy body, organized but deprived of sensibility; it is generally softish, but at other times more or less hard; its form is various and indeterminate: it originates and is developed within the uterus, and after a while is expelled from its cavity." The author details the very various opinions of the old medical writers upon the origin and nature of these substances; and believes himself that, in most cases, they are blighted ova; but as they have been also repeatedly met with in old women, in women whose chastity was beyond suspicion, and in others, in whom the hymen continued perfect, he adds, that they undoubtedly may occur independently of conception, and, in such cases, supposes them to arise from the organization of a clot of blood thrown out into the cavity of the uterus. The embryo is frequently dissolved and removed, and the mole is then a placentar-looking substance, having a cavity containing more or less fluid in its substance, and lined with a serous membrane. When the fluid has been discharged prior to its expulsion, the mole becomes solidified and indurated, while traces of cavity can only be discovered by careful examination; and the existence of which at all may sometimes be doubted. When the fœtus has died at a more advanced period of gestation, portions of its osseous structure may be found in the substance of the mole. Moles possess a mere vegetative life, and no true circulation. Sinuses on their surfaces communicate with those of the uterus, and frequently long prior to their expulsion they give rise to exhausting hæmorrhages. More than one is sometimes found. A mole may exist prior to conception, and is sometimes found with a full-grown fœtus. Coagula of blood may exactly resemble moles until cut into. The mole is usually expelled in 60 or 80 days, and great suffering and hæmorrhage may be then present. The lochia and condition of the breasts are much the same as after natural labour. The period of expulsion, age, and very existence of a mole is usually a mere matter of conjecture, as the diagnostic marks are utterly insufficient. When it complicates pregnancy, the fœtus, deprived of due nutriment, usually perishes, and premature labour results. The ancients, when they supposed these bodies to be present, adopted the most injurious practices, such as violent medicines and exercises, in order to dislodge them. The moderns leave the case to nature, only interfering when hæmorrhage, imperfect expulsion, &c. demand their doing so.

*Physometra, or Uterine Tympanitis.*—The air producing this affection generally proceeds from some body putrefying within the uterus, the occlusion of its os by spasm, coagula, mucosities, or other mechanical cause preventing the escape of the gas, which frequently consists of sulphuretted hydrogen. Nervous women who have borne children are most liable to this complaint. The distended uterus may rise even above the umbilicus, and the pressure it produces gives rise to much pain and suffering, and sometimes to great constitutional irritation. Temporary relief may be obtained by the partial discharge of the air; but, mostly, the discharge occurs suddenly and with violence, and the patient is at once cured. The diagnosis from pregnancy, owing to the lightness and elasticity of the tumor, is usually not difficult. As inertia of the womb is generally the cause of the affection, tonics and general or local excitants are useful, but injections into the cavity of the womb itself are dangerous. The escape of the air may be facilitated by the introduction of a tube into the os uteri. In strong women, when sub-inflammatory action may be supposed to be present, small bleedings, warm baths, cataplasms, lavements, &c. must be employed; and, in most, a small derivative bleeding, providing great debility be not present, is serviceable. The entrance of atmospheric air from without, and its dilatation of the uterine cavity, believed by some, is very doubtful.

*Œdopsophia, or Noisy Expulsion of Gas from the Vagina.*—This affection occurs in women (especially lame women) whose inferior vulvar aperture is narrow. Oftentimes no symptom precedes it, and the patient is not aware of the existence of the complaint until the noisy expulsion occurs. The distended state of the parts may be felt by examination by the rectum, and the gas in part expelled by pressing on the hypogastrium. When the affection arises from a foreign body in a state of putrefaction, it will cease on the removal of this; but, in instances where none such existed, Lisfranc has frequently rendered the patient's condition much more supportable, by instructing her, prior to going into society, or, under any circumstance where the unexpected expulsion would prove annoying, to secure herself from this, by introducing a finger into the inferior aperture of the vulva, and thus give vent to it. In examining females for other complaints, he has frequently detected this affection, although concealed by the patients, and has always succeeded in affording great comfort by the above suggestion. If the vagina is capable of secreting this gaseous substance, it is from its being in a condition of inflammation or irritation, and therefore means of a mild antiphlogistic character, modified by circumstances, must be had recourse to, *e. g.* venæsection, warm baths, lavements, &c.

*Hydrometra, or Dropsy of the Womb.*—This is a rare affection, generally occurring during gestation. Sometimes the fluid escapes in small quantities by degrees, and, more rarely, the whole disappears at once to be shortly reproduced. The affection does not seem one that is immediately dangerous in itself, especially if the fluid escape from time to time: but the debility, and bad constitution of the subjects of it, and the organic changes in the womb or other viscera, which produce the affection, may give rise to serious cause of alarm. Occasionally, the fluid is removed at the third or fourth month of pregnancy, and is not reproduced; or it may finally disappear during delivery. In treating this disease we must look upon it as a mere symptom of some uterine affection. But there are cases where the presence of the fluid neutralizes the means adopted, and, if the symptoms are urgent, it must be evacuated. The use of violent aperients, the injection of substances into the womb, the employment of local or hip baths, and all similar means, tending to produce congestion of the uterus, must be interdicted. Secale cornutum, which has been recommended, has in some cases led to fatal inflammation. When the patient is not too feeble, and congestion exists, revulsive bleeding, general warm baths of bran-water, emollient injections, moderated exercise, and careful diet, are appropriate means. While inflammation is present, we should not empty the cavity, unless some urgent symptom exist, when the finger or a catheter may be introduced into the os uteri.

*Inversion of the Uterus.*—This may be complete or incomplete; and in these cases in which complete inversion has been thought to occur sometime after delivery, it probably had existed in an incomplete manner from the beginning. As dilatation and softening of the uterine walls are only required for its production, it is found, not only after delivery, but also in women who have never borne children—*e. g.*—after the sudden removal of a polypus from the fundus uteri; and the affection may be produced, not only by traction, but by the compression of the upper portion of the enfeebled walls of the uterus, by the weight of the intestines, and the contraction of the abdominal muscles.

It is surprising how little suffering an affection, usually so formidable, produces in some cases. The author relates a case of an old woman who died of bronchitis in the Salpêtrière, and upon the examination of whose body the most complete inversion of the uterus was found. Convalescent from a former attack, she had been under observation a considerable time prior to the fatal seizure of bronchitis, and was observed to be very active in her habits, regular in most of her functions, and manifesting no one symptom whatever of uterine derangement, and the inversion was discovered after death, as it were, by accident. As a general rule, however, those women who do not perish forthwith drag out a miserable existence. M. Lisfranc does not believe in the authenticity of those cases, in which the uterus is said to have been reduced spontaneously or by art, at the expiration of days, weeks, or even months after the inversion has occurred.

*Prolapsus of the Uterus.*—Upon the effect of *engorgement* or hypertrophy of the uterus in inducing this, the author thus remarks.

"Displacements of the uterus are astonishingly frequent. I have demonstrated this fact too frequently to my clinical class at La Pitié for it to be doubted. But it is generally believed that these affections are almost always essential (primary). I am not of this opinion, and it has caused grave errors in therapeutics to be committed; for, on admitting it, the descent, or deviation of the womb is alone treated, and the uterine engorgements are neglected until they become incurable. I have advanced elsewhere that I will prove, whenever it may be required, that the descent, prolapse, anteversion, retroversion, and lateral inclination of the uterus, are excessively rare, when this organ is exempt from hypertrophy. For more than fifteen years I have especially directed my attention to this important point of pathology. I have examined thousands of women, and, to the present time, I have found some few cases only in which these morbid affections existed without a sensible increase in the size of the uterus. * * * * When the uterus is engorged in its entire circumference it descends parallel to the pelvic axis: if its increase of volume prevails in front there is anteversion, and the contrary when such augmentation occurs at the posterior portion of the organ. Lastly, when the induration exists upon one side, to that side it inclines. It requires but the simplest knowledge of physics to perceive that a pyriform body, somewhat flattened, suspended in the pelvis by four supports, must, if its anterior portion acquires a considerable thickness, execute a movement which will carry its superior portion towards the symphysis pubis, and *vice versâ.*

"But I have often heard the following question asked by men who object to these new ideas. 'Is this hypertrophy, which is observed in displacements of the uterus, primary or consecutive?' If it followed the displacements it is evident we should very frequently meet with these affections alone, for, before the development of the engorgement, the patients would suffer pain, and in examining them we should find such engorgement absent; but essential displacements being in fact exceedingly rare, every one must admit their production by the hypertrophy. If required, there is still another proof in favour of this opinion. The engorged womb is displaced—I confine my treatment simply to the engorgement. I cure it, and the organ shortly after resumes its natural position in the pelvis.

"These statements are neither idle nor merely curious. Every one, reflecting upon the subject, must see that it is not a matter of indifference whether we exclusively treat any displacement of the uterus, or whether we attack an engorgement of that organ." 409-11.

The engorgement, in fact, must be the primary object of treatment, and, if degeneration of structure has not occurred, may often be effectually relieved. The pessary must be delayed until this is subdued, and may not even then be required.

In reference to the irritation of the bladder, so frequently prevailing when the uterus is displaced, M. Lisfranc observes—

"This inconvenience is especially found in pregnant women. An advantageous means of facilitating the expulsion of the urine is to pass the finger into the vagina behind the pubes, and thus relieve, for the time, the neck of the bladder and urethra from the pressure which the uterus exerts upon them. The patients easily perform this manœuvre for themselves.

"Women are also often tormented by excessively frequent desire to urine, so that, especially about the menstrual periods, they have to rise sometimes 15 or 20 times in a night. Topical emollients and narcotics occasionally produce excellent effects. I have great confidence in the following remedy, founded upon the great success that has attended my use of it, conjoined with derivative bleeding from the arm. A small lavement, nearly cold, is administered night and morning, containing a few grains of camphor, suspended in yolk of egg, and a few drops of Sydenham's laudanum." 429.

*Retroversion of the Uterus.*—This is more common than the prolapse, but not so much so as the anteversion of the organ. Its usual source is engorgement, and it may occur suddenly or come on slowly. Pelvic tumors often cause or increase the displacement; they can be traced in contact with the uterus, and yet are distinct from it, as the non-effacement of its cervix proves among other marks. The replacement of the uterus is painful and difficult, and yet not permanent. When it occurs during pregnancy, the development of the womb is often interfered with, and abortion may be produced. It may exert most injurious pressure upon other organs, and especially upon the bladder, giving rise to retention of urine, difficult frequently of relief, and yet, if not relieved, leading to gangrene or rupture of the bladder. Violent tenesmus is also present, and the woman is tormented with most urgent expulsive efforts, sometimes forcing the womb far beyond the external parts. Before any attempts at reduction are made, all inflammatory action must be relieved, when, indeed, the organ will frequently return to its normal position. Where this is not the case, the author furnishes us with the mode of procedure for effecting the reduction.

*Anteversion of the Uterus.*—In this (the converse of the preceding) we have the fundus of the uterus directed towards the pubis, pushing the bladder downwards and forwards, and its cervix lodged in some portion of the concavity of the sacrum. It is usually said to be less frequent of occurrence than retroversion, but this is only the case when the uterus contains the fruit of conception. In the empty state of the organ the deviation now under consideration is infinitely more common. The anterior face of the womb is more exposed to external injury, and consequent congestion. The habit women have of frequently emptying the bladder, also, allows the uterus, already disposed to do so by its weight, to descend in front. The habitual constipation of the Parisian women is another cause—the fæces being detained, as may be felt on examination, for some time opposite the junction of the neck and body of the uterus.

True anteversion is very rare in pregnancy. The symptoms and examination, when carelessly made, have sometimes led to the belief of the existence of an affection of the bladder rather than one of the uterus. This displacement usually comes on slowly, is less complete and less serious than retroversion, for its reduction is much easier.

*Inclination or Obliquity of the Uterus.*—This may arise congenitally from faulty conformation of the pelvic cavity, from constipation retaining the fæces above the cervix uteri, pelvic tumors, unequal length of the pelvic ligaments, &c.; but, when it occurs independently of pregnancy, it is usually caused by lateral engorgement of the uterus, or the presence of fibrous tumors. The author recommends early manual interference for the purpose of directing the os uteri towards the centre of the pelvis, and the due attention to the position of the woman during pregnancy.

*Means of counteracting Displacements and Engorgements of the Uterus.*

*Sponges.*—These must not be employed until all inflammation and pain are subdued, and, even when these are absent, they sometimes cause great suffering, compelling their removal, although the woman may have kept very quiet, and taken narcotics. But they frequently occasion great inconvenience at first, and yet must not too hastily be withdrawn; for the patient, becoming shortly accustomed to them, ceases to suffer. Indeed, neuralgic pains are often dissipated by the sponge, and it may sometimes be employed with advantage when we are in doubt as to the exact nature of the pain which exists. It must be frequently removed and cleansed, or it will give rise to inflammation and ulceration of the parts with which it is in contact. Sponges are useful in a relaxed state of the uterus, or of the utero-vulvar membrane, and in the early stages of the uterine displacements, but are quite inoperative against complete prolapse.

*Pessaries.*—Many women have the greatest repugnance to the employment of these, so as to render delay in their use imperative. Although some painful and irritable conditions of the vagina may be relieved by pessaries, as a general rule they become aggravated by them. In cases where no suffering pre-existed, very much is induced by these instruments. The existence of an abundant leucorrhœa, when not of an inflammatory origin, does not contra-indicate their use.

After the first application of the pessary the female should observe the recumbent posture for five or six days or longer, lying on a sofa, or long chair, preferably to a bed, the heat of which produces congestion of the genital organs. Exercise must be only very gradually resumed. When the instrument excites great irritation, some blood should be abstracted, emollient injections and anodyne lavements administered, light diet adhered to, and a luke-warm anodyne cataplasm laid upon the belly. If the irritation does not subside, the instrument must be removed.

The pessary should be removed every 15 or 20 days, cleansed, and re-applied immediately; and when much suffering results from its use, we should carefully examine, to ascertain whether this arises from its having shifted its proper position, so as to require re-adjustment. The author enters into long detail of the different varieties of pessary.

Pessaries, which have been allowed to remain in the vagina for months or years, sometimes become so impacted, as to require dangerous operations for their removal. At other times, they penetrate the walls of the vagina or rectum, and excite acute inflammation or typhoid fever. When they excite inflammation in the textures they are in contact with, their removal is called for; but the question arises, whether this should be effected at once. This was M. Lisfranc's practice at the commencement of his career; but, having found the process of removal much aggravate all the sufferings of the patient, he subsequently put into force the antiphlogistic treatment prior to attempting it. When the pessary projects much towards the rectum, as it so frequently does, it is best removed by manœuvres made within the gut. When pessaries cannot be borne, small bags may sometimes be introduced into the vagina and subsequently inflated. Frequently the patient is cured, and the negligence of her attendant prevents his ascertaining that the pessary is no longer required, and thus it is often needlessly and injuriously retained.

*Redness, Pimples, Aphthæ, and Granulations, situated on the Cervix Uteri.*—The posterior lip of the os uteri is especially liable to these. Patches of redness, separate or confluent, are often found, especially when uterine leucorrhœa is present. Such redness is frequently found at the periods of puberty, and of the cessation of the menses; and is distinct from the normal redness observed in pregnancies and during the catamenia. Frequently no distinct symptoms are present, and it is not until the speculum is employed that a condition of parts, capable of leading to much mischief, if not relieved, is discovered. Neglected, incurable ulcers may follow. Antiphlogistic treatment is first required, and that proportioned to the acute or chronic character of the accompanying inflammation. Afterwards the fluid protonitrate of mercury should be applied, in the slightest possible manner, by means of a camel's hair pencil, to a small part only of the diseased surface. The speculum should then be filled with nearly cold water to counteract the effect of any superfluous application that may have accidentally resulted.

*Non-Cancerous Ulcers of the Uterus.*—These are very common in Paris, where leucorrhœa is endemic; they present every variety as regards form, size, and number, while their chief locality is the posterior lip of the os uteri. Hypertrophy accompanies and precedes the affection.

Sometimes the ulcers give rise to hæmorrhages, which must be met with depletory or derivative bleeding, and the application of the protonitrate of mercury, as varicose tumor will become developed if the case is neglected. The ulcers are frequently of very slow and insidious progress. They are frequently mistaken for cancer, offering then scarcely any diagnostic mark beyond that derived from the success of treatment. This consists of antiphlogistic and soothing means until active inflammation has become dissipated. Injections of various kinds may then be used, and the iodide of potassium administered. In this way ulcers of bad aspect may often be cured, as in other parts of the body, without having recourse to caustics. But as a general rule these are necessary for the treatment of ulcers of these parts, but they should usually be deferred until all active irritation has become subdued. Still, if the ulcer makes great progress, in spite of antiphlogistics, which is not usual, or even remains stationary for two or three weeks, the use of the caustic must not be delayed. The fluid protonitrate of mercury is to be applied, rather with the intention of modifying the vitality of the part than of producing its disorganisation, except in the cases of very deep ulceration, extensive fungosities, or suspected cancer, when its application must be more extensive. The cauterization requires frequent repetition. When the ulceration is very slight, or mere excoriation, the nitrate of silver will suffice, but this sometimes causes hæmorrhage from the sore. From three to six months are generally required for the treatment of these cases, at other times much less time; while again some require even years. Out of 50 cases, the author states he has cured 42, by cauterizing in this way; but some are so obstinate as to wear out the patient by the discharge and excessive pain they cause, and render their removal by the knife imperative.

*Cancer of the Uterus.*—Cancer of the uterus without ulceration is rare, for in most cases it commences by ulcers, which at first appear quite simple. Still, occult cancer is occasionally met with, may be indolent, and frequently presents no symptoms distinctive from other engorgements. It must be treated as they are, and when suspected to be cancer, care must be taken not to practise any opening into its substance.

M. Lisfranc delivers his opinion, as to the *age* at which cancer usually occurs, in the following terms, in which he will be found to differ from other observers.

"Cancer is not found equally prevalent at all ages. It has been said that this disease usually manifests itself between the 40th and 45th years, and that then it became less frequent in the following progression: from 30 to 40; from 45 to 50; from 20 to 30; from 15 to 20; from 50 to 60; and, lastly, from 60 to 70. The numerous women I have attended since 1815, have convinced me that cancer of the uterus attacks most especially between the ages of 18 and 35, confirming the great principle of pathology, that an organ is more liable to become diseased in proportion to the activity with which its functions are carried on. It is erroneous to believe that this disease is more common at the period of the cessation of the menses. I have seen a cancer occupying the cervix uteri of a young girl in her 15th year. From 70 to 80 years of age uterine cancer is very rare." 610.

On the importance of early examination, the author thus expresses himself.

"It is, unfortunately, not rare to meet with a great number of women, in whom cancer has become incurable, without even its existence having been suspected. Is it a fact, that it is sometimes entirely latent? The mere idea of the affirmative of this, would carry consternation into every family. But fortunately it is not the case. I am convinced, that among the thousands (the author constantly uses this numeral when alluding to his cases) of cases which I have observed I have never yet seen one in which a morbid affection of the uterus, of whatever description it may be, did not manifest itself by easily appreciated symptoms. Thus, leucorrhœa, more or less permanent, anomalies in the menstrual function, lumbar, and other pains, &c. suffice to excite the attention of the well-informed practitioner. He does not forget that the slightest inconveniences originating in, or appearing to do so, affections of the genital organs imperiously require that manual examination, and that by the speculum, should at once be put into force. Since these wise precepts, so much insisted upon by Leuret, have been revived, since women, of all classes of society, have received salutary warnings from others, who themselves have benefited by a timely foresight, we have seen many fewer cases of incurable diseases of the uterus. Some time since, eight or ten women would come every week to La Pitié, in whom cancer of the uterus had proceeded beyond the resources of art, scarcely any of these unfortunate beings having been submitted to examination. At present we scarcely meet with two or three in a month. Let us hope that their number will continue to decrease; and we shall feel too happy if we have been able to contribute to its doing so." 613.

The author frequently alludes to the peculiar *odour* of cancer, and, in some doubtful cases, considers it may aid the diagnosis.

"The retention of coagula and secretions in the vagina, may produce most disagreeable and disgusting odours; but they do not resemble that of cancer, which is *sui generis*, and which cannot deceive the clinical observer. It is yet more impossible to confound it with gangrenous miasmata. It is tainted (infecte) and nauseating, and distresses the patients themselves; while it oftentimes penetrates so far into the apartment, as to render it nearly uninhabitable, in spite of efficient ventilation." 621.

The internal use of the iodide of potassium, and the external application of the protonitrate of mercury, are often of great service in cases apparently desperate; and in most they procure at least alleviation and retardation.

*Amputation of the Cervix Uteri.*—This may be performed—1. When the cancer is well characterised, and extends too deeply to admit of the use of caustic. 2. When it does not extend above the superior portion of the uterine insertion of the vagina. 3. As some ulcers of the leg require amputation, so may ulceration of the cervix, though not carcinomatous, justify the operation when, from its utter obstinacy, it proves too much for the debilitated condition of the patient. 4. The fact of the uterus being in a state of hypertrophy or engorgement must not necessarily prevent the operation. 5. The performance of the operation is encouraged by the fact, stated by Bayle, and confirmed by the author, that cancer of the uterus is attended with far less implication of surrounding parts, or affection of the absorbent glands, than when it attacks other organs. 6. As in cancer in general, it is but a limited portion of the tumefaction which is specifically diseased. 7. Even very great increase in the size of the ovaries must not necessarily prevent the operation.

8. Where the whole of the diseased portion cannot be removed, that which remains may be cauterised. But the author states that very indifferent success usually attends this, which he calls the mixed operation.

9. It is an error to suppose that much pain or hæmorrhage attends the operation. Many patients at its completion are not aware that it has been commenced, while the bleeding is often very inconsiderable and easy of arrest.

The operation does not prevent future pregnancy, and not only does the fœtus usually reach its full time, but the labour is terminated with greater facility: of twelve persons who became pregnant, one only miscarried, and this was attributable to her own imprudence. In one case, twins, full grown, were easily delivered.

In one, only, among his numerous cases, has the author known the cicatrix produce obliteration of the os uteri.

Throughout his work, our author manifests a morbid sensibility as to the opinions of others concerning his statements, and a disposition to charge upon them the purloining of his discoveries and improvements in medical science. Upon the subject of amputation of the cervix uteri, he is especially sore. Our readers are aware, that the authenticity of his cases has been very generally disputed, both in France, and in this country; and that he has been charged, not only with performing the operation where no cancer whatever existed, and reporting it as cancer cured, but also with very much understating the amount of the mortality which has attended his proceedings. His defence in the present work is lame beyond conception. Instead of meeting his antagonists upon the questions of fact which they have adduced, he charges them with having obtained his documents (which, strange to say, he had himself prohibited the publication of,) surreptitiously from the Academy, and travestied their contents. He states, that, in his paper upon the subject, he merely declared that after 99 operations, 84 patients were cured, and 15 only died; but that he declines all verifications of such astounding results. "Professional honour imperiously forbids my doing so. I would not at any price betray the secrets of families. I will never foget the oath of Hippocrates." In answer to such evasive nonsense as this, we can only say, that M. Lisfranc has much over-rated the authority he supposes himself to possess in the medical world, if he thinks he can introduce an operation of this character into general use, without demonstrating, not only the ease with which it may be performed, but also the success that attends it; and that, not by mere assertions, but by authenticated facts. It is instructive to learn that he seldom now performs it himself. It is true that he explains this by the fact, that cancerous diseases are now so well ascertained, and treated in their earlier stages, (by the means he has introduced) that cases calling for operation comparatively seldom present themselves. The melancholy truth, however, forces itself upon our conviction that he has been deluded by the very ignorance of diagnosis he so bitterly blames in others, or by mere temporary success, into the performance of many most unnecessary and unjustifiable operations, until his rashness has been restrained, and he himself forced upon a more legitimate mode of proceeding, by the indignant voice of that profession, which would have been but too well pleased to have followed his practice, and participated in his success, had the one been rational, and the other assured. Who is there believes, that the number of cases of *genuine cancer of the uterus* has been diminished materially by improvements in the mode of treatment of the disease?

*Affections of the Fallopian Tubes and Ovaries.*—One or both of the Fallopian tubes may become obliterated, sometimes through the whole extent of the canal, but usually only in limited portions of it. The affection may be congenital, or produced by compression, coagula, retained secretions, &c. It may result also from inflammation extending itself from the uterus or peritoneum. Sterility has often been observed in women who prior to marriage had been affected with metro-peritonitis, or who have subsequently suffered from it after accouchements; and by employing means adapted to chronic or acute inflammatory action, the author has frequently been enabled to relieve this condition. Inflammation of the ovaries sometimes follows a laborious accouchement, metro-peritonitis, &c.; and, when not proceeding from some such cause, it is a rare and usually subacute affection, very insidious in its progress and sometimes brought on by cold, emmenagogues, the abuse of sexual intercourse, &c. Antiphlogistics and the iodide of potassium are the chief remedies.

*Magnetism.*—Mr. Ward of Ollerton and his lawyer-operator must not imagine themselves the introducers of magnetism as an agent in surgery. Our author has the following passage.

"Many affections of the womb produce violent sufferings, which even the muriate of morphia introduced by means of a blister fails to relieve. These pains, which manifest more or less of a neuralgic character, are remittent or intermittent, their exacerbations produce in the patient a state amounting to desperation, leading her sometimes into the greatest danger. The physician, finding he has exhausted the resources of his art, remains a mere spectator of these dreadful scenes whose termination cannot be foreseen. There is, however, a powerful means to which you must then necessarily have recourse, viz. magnetism. Far be it from me to admit the reveries of the magnetizers; but it is quite certain that Mesmerism produces a most extraordinary effect upon the nervous system of the women we are now alluding to. I have convinced myself of this a great number of times. I have seen the pains dissipated as if by enchantment." 725.

This volume, as we have said, concludes the present work; but others upon Operative Medicine are to follow.

